# The use of tDCS as a therapeutic option for tinnitus: a systematic review^☆^

**DOI:** 10.1016/j.bjorl.2018.02.003

**Published:** 2018-03-09

**Authors:** Amanda dos Humildes Maia Santos, Afonso Paranhos Silva Santos, Henrique Souza Santos, Adriana Campos da Silva

**Affiliations:** a Escola Bahiana de Medicina e Saúde Pública, Medicina, Salvador, BA, Brazil; b Pesquisa da Liga Acadêmica de Neurocirurgia, Salvador, BA, Brazil; c Hospital Geral Roberto Santos, Neurocirurgia, Salvador, BA, Brazil; d Grupo de Ensino e Pesquisa em Endocrinologia (GEPEN), Salvador, BA, Brazil; e Linha de Pesquisa “Comportamento e Aprendizado Motor”, Salvador, BA, Brazil; f Grupo de Ensino em Neuroanatomia, Salvador, BA, Brazil; g Escola Bahiana de Medicina e Saúde Pública, Fisioterapia, Salvador, BA, Brazil; h Universidade Federal da Bahia (UFBA), Gerontologia, Salvador, BA, Brazil; i Universidade Federal do Rio de Janeiro (UFRJ), Ciências Morfológicas, Rio de Janeiro, RJ, Brazil; j Universidade Federal da Bahia (UFBA), Ciências da Saúde, Salvador, BA, Brazil

**Keywords:** Tinnitus, tDCS, Transcranial direct current stimulation, Zumbido, tDCS, Estimulação transcraniana direta

## Abstract

**Introduction:**

Due to the subjectivity of the tinnitus diagnosis and its diverse etiologies, establishing an effective treatment is complex. In this context, transcranial direct current stimulation, a noninvasive option, is available for most patients and has shown good results in the treatment of other symptoms such as chronic pain.

**Objective:**

To evaluate the therapeutic response of tinnitus to transcranial direct current stimulation.

**Methods:**

A systematic review of the literature was performed using the following descriptors: tinnitus, transcranial direct current stimulation and randomized clinical trial. The research was carried out in the MEDLINE/PUBMED, Lilacs, and Scielo databases. The inclusion criteria were: patients over 18 years of age with no associated comorbidities, who had a diagnosis established by a specialist or through the application of previously validated scales and criteria applied by a non-specialist physician.

**Results:**

A total of 4165 studies were found, and a total of six were selected after the inclusion criteria were applied, obtaining a sample of 602 patients. Based on the defined criteria, there was a positive response to transcranial direct current stimulation in 14.86% of the participants.

**Conclusion:**

Based on literature studied, there is no therapeutic response of tinnitus to transcranial direct current stimulation.

## Resumo

**Introdução:**

Devido à subjetividade do diagnóstico do zumbido e a suas diversas etiologias, o estabelecimento de um tratamento eficaz é complexo. Neste contexto surge a *transcranial direct current stimulation*, uma opção não invasiva, acessível para grande parte dos pacientes, e que tem apresentado bons resultados no tratamento de outros sintomas como dor crônica.

**Objetivo:**

Avaliar a resposta terapêutica do zumbido ao *transcranial direct current stimulation*.

**Método:**

Foi realizada uma revisão sistemática da literatura, por meio dos seguintes descritores: zumbido, *transcranial direct current stimulation* e ensaio clínico randomizado. A pesquisa foi realizada nas bases de dados MEDLINE/PubMed, Lilacs, Scielo. Os critérios de inclusão foram: pacientes maiores de 18 anos sem outras comorbidades associadas, que tiveram diagnóstico estabelecido por um especialista ou por meio da aplicação de escalas e critérios previamente validados aplicados por médico não especialista.

**Resultados:**

Foram encontrados 4.165 estudos, sendo selecionado após a aplicação dos critérios de inclusão um total de seis, obtendo-se uma amostra de 602 pacientes. A partir dos critérios definidos, houve uma resposta positiva ao *transcranial direct current stimulation* em 14.86% dos participantes.

**Conclusão:**

Baseada na presente revisão, não há ainda uma resposta terapêutica do zumbido ao *transcranial direct current stimulation*.

## Introduction

Tinnitus can be characterized as an auditory illusion, i.e., a sound perception unrelated to an external source of stimulation. It affects both sexes equally, occurring more frequently between 40 and 70 years of age.^1^, ^2^, ^3^ It affects approximately 40 million people, both in the United States and England, and is considered the third worst problem that can affect the human being,^2^ reducing the quality of life of the population around 1%.^4^ Due to the initial difficulty in determining the etiological diagnosis, the treatment cannot be easily established, often concentrating only on symptom reduction.^1^, ^4^

The etiology of tinnitus has several classifications in the literature, and the most frequently used one, due to its simplicity in differentiating of the origin of the tinnitus, is the one that differentiates tinnitus from para-auditory structures, usually of muscular or vascular origin, from the neurosensory auditory structures.^5^ Tinnitus resulting from the sensorineural auditory system can have several causes: otologic, cardiovascular, metabolic, neurological, pharmacological, odontogenic and psychogenic.^5^, ^6^

Regarding treatment, there is no clinical evidence establishing one type of treatment as the ideal one. However, one should always treat tinnitus symptoms and, particularly, seek for a structural cause, such as those already mentioned. If there is no obvious structural cause and the patient has a persistent tinnitus, some drug and non-drug therapy options are available.^6^

It is in this context that Transcranial Direct Current Stimulation (tDCS) is recommended as an alternative treatment to decrease the perception of tinnitus. tDCS is a non-invasive form of transcranial stimulation, in which the electrical current passes through the cerebral cortex through electrodes implanted on the skin.^6^ Due to its non-invasive, low-cost, easy-to-apply and pain-free characteristics, in addition to less residual inhibition when compared to other stimulation methods, tDCS is an intriguing choice of method for tinnitus treatment.^6^, ^7^ However, there is no consensus on precise parameters for its use in this specific condition. This review, therefore, brings together the results of clinical trials on symptom improvement, with the objective of analyzing the therapeutic response of tinnitus to tDCS and possible differences observed, depending on the location and type of electrode.

## Methods

### Type of study: systematic review

Ethics committee approval: Because it was a systematic review, there was no need for it.

#### Inclusion criteria

Randomized clinical trials (RCTs), quasi-randomized clinical trials, and open-label studies of patients older than 18 years were included. We considered as RCTs the ones that performed randomization using coins, data or randomization performed by the computer. Studies using other forms of randomization were classified as quasi-randomized.

Studies in which tinnitus was diagnosed through a specialist's evaluation or the use of validated scales and criteria for the symptom and that evaluated the use of tDCS as a therapeutic option for tinnitus, with the control group using a placebo stimulus were included. The study should have used tDCS for at least two sessions, and any symptom improvement was considered an attenuation of the intensity and discomfort caused by tinnitus, as well as other associated symptoms.

#### Exclusion criteria

Other types of study were excluded (case–control, cohort, case reports), as well as studies with populations younger than 18 years, without the presence of a placebo control group or studies that used another type of therapy associated with tDCS. Furthermore, studies including patients with comorbidities associated with tinnitus symptoms (previous head trauma, use of ototoxic substances, Meniere's disease, epilepsy, intracranial tumor) or studies that used only one tDCS session were excluded.

#### Electronic search

The search for articles was carried out in the MEDLINE/PUBMED, Lilacs, and Scielo databases, without restrictions on year or language of publication. The following key words were used to identify relevant articles in the electronic databases: tinnitus, tDCS, randomized clinical trial and their respective Portuguese correlates, in addition to the use of the Medical Subject Headings (MeSH).

#### Study selection

The search and analysis of articles occurred from 09/01/2016 to 09/25/2016. Initially, articles in duplicate were verified using database management, Microsoft Office Access 2013. After this step, articles selected only through title and summary were evaluated according to the eligibility criteria, excluding irrelevant articles. The selected articles were read in full to decide on their inclusion. Subsequently, the researchers separately performed the data extraction, and after the data collection, a new consensus meeting was held to verify the degree of agreement between the authors. When disagreements occurred at the consensus meetings, the third researcher (A.H.M.S) was utilized to reach an agreement.

To evaluate the quality of the selected articles, the Consolidated Standards of Reporting Trials (CONSORT) checklist was applied and, to evaluate the final quality of the systematic review, the Preferred Report Items for Systematic Reviews and Meta-Analyzes (PRISMA) checklist was applied. Moreover, an active search was carried out for other studies mentioned in the references of the selected articles to be added to the literature review.

#### Data extraction

The data of the selected articles were collected using a predefined form with the following information: title, author (s), year of publication, country of origin, language, keywords, objectives, study design definition, method, sample size, diagnostic criteria for tinnitus, time of treatment with tDCS, characterization of participants, mean age and gender.

Finally, the COCHRANE and PROSPERO databases were searched for similar reviews, so there would be no duplicate information.

## Results

### Study selection

**Figure 1:**
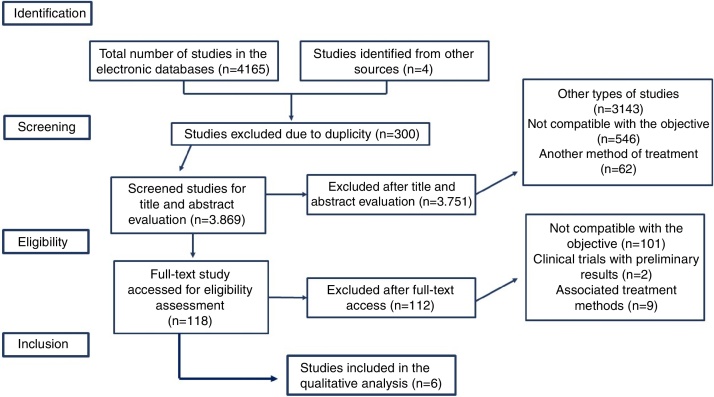
Study selection algorithm.

In the first stage, a total of 4165 studies were identified in the electronic databases, according to the defined search strategy. Three hundred were excluded because they were duplicates and 3108 were excluded through the “clinical trial” filter. Of the remaining 658, 643 were excluded, after title and abstract analysis, at the screening stage. Of the excluded articles, 546 (85%) were not compatible with the study objective, 67 (10.5%) used another treatment method and 30 (4.5%) were review studies. Among the ones that were not compatible with the study objective, there were articles that addressed other types of neurostimulation and pharmacological methods. Among the 114 studies selected at the screening stage; 99 (95.19%) were not compatible with the study objective; 1 (0.96%) was characterized as a clinical trial with preliminary data only; 1 (0.96%) scored < 40% on CONSORT, 4 (3.84%) used other associated treatment methods and, finally, 3 (2.88%) were duplicates. Thus, 6 (0.14%) studies were selected at the eligibility stage. The steps followed for study selection are shown in Fig. 1, detailing the inclusion and exclusion criteria used. Table 2 describes the overall study characteristics. The result of the qualitative evaluation of the studies using the CONSORT checklist is described in Table 3.

**Table 1:** Inclusion and exclusion criteria.

Inclusion criteria	Exclusion criteria
Randomized, quasi-randomized clinical trials and open-label trials.	Case–control, cohort studies, case reports.
Patients older than 18 years.	Patients younger than 18 years.
Tinnitus diagnosed by specialist assessment or through scales validated for the symptom.	Tinnitus-associated comorbidities: history of head trauma, epilepsy, use of ototoxic substances, intracranial tumor, Meniere's disease.
Use of tDCS for at least two sessions and no other associated therapy.	Use of tDCS in only one session.

**Table 2:** General data from studies included in the systematic review, ordered by year of publication.

References	Study type	Sample size	Stimulation site	Type of stimulation	Time of stimulation (minutes)	Intensity (mA)
Vanneste et al.^6^	OL	478	DLPFC	Anode on the right and cathode on the left/anode on the left and cathode on the right	20	1.5
Garin et al.^9^	RCT	20	LTPA	Cathode or anode on the left	20	1
Frank et al.^11^	OL	32	DLPFC	Anode on the right and cathode on the left	30	1.5
Faber et al.^8^	RCT	15	DLPFC	Anode on the right and cathode on the left/anode on the left and cathode on the right	20	1.5
Forogh et al.^10^	RCT	22	LTPA	Anode on the left and cathode in supra orbital area	20	2
Pal et al.^7^	RCT	42	AC/DLPFC	Cathode on the auditory cortex and anode on the DLPFC	20	2

OL, open label; RCT, randomized clinical trial; DLPFC, dorsolateral pre-frontal cortex; LTPA, left temporoparietal area; AC, auditory cortex.

**Table 3:**
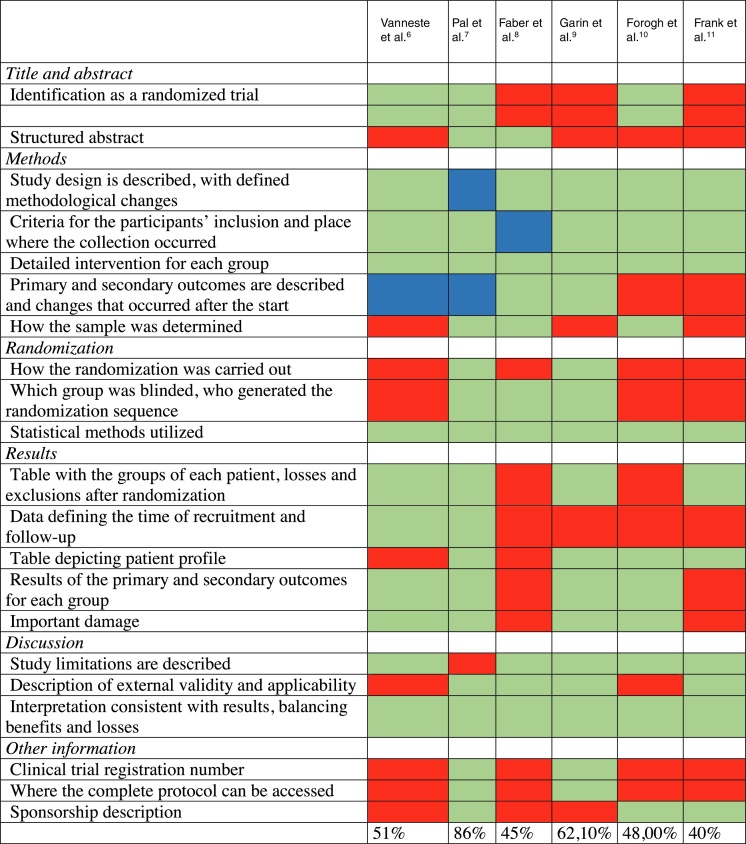
Quality evaluation of the selected studies, based on the essential items of CONSORT.

Markings in red, did not answer; markings in blue, partially answered; markings in green, answered.

### Scales used to assess the primary endpoint

Among the selected studies, 3 (50%) had as objective to evaluate the improvement in the intensity and the annoyance caused by tinnitus, using the visual analogue scale (VAS). Only one study aimed to evaluate its primary outcome through the clinical global impression (CGI) scale. Two studies (33.3%) had as their primary endpoint the assessment of the tinnitus severity degree using the tinnitus handicap inventory (THI).

### Therapeutic response of tinnitus to tDCS

Among the studies that used the VAS scale to measure the response to the symptom, all (100%) had at least one statistically significant result. The study by Vanneste et al.^6^ showed a significant response when the electrode was implanted with the anode on the right and the cathode on the left, with an improvement in both intensity and stress caused by tinnitus (*p* < 0.001*). When implanted with the anode on the left and cathode on the right, there was no improvement in any of the scales (*p* > 0.05). Pal et al.^7^ used the THI scale as the primary endpoint, obtaining non-statistically significant results (*p* = 0.69).

The study by Faber et al.^8^ showed an improvement in the VAS scale on annoyance (*p* < 0.05*), using the electrodes implanted with anode on the left and the cathode on the right. The study developed by Garin et al.^9^ showed a statistically significant response in the VAS scale for intensity (*p* = 0.013 * for the cathode and *p* = 0.020* for the anode) and non-significant for the discomfort (*p* = 0.108 for both stimuli).

The study by Forogh et al.^10^ used the CGI scale as the primary endpoint and also obtained a non-statistically significant result (*p* = 0.807). Finally, the study by Frank et al.^11^ also used the THI scale as the primary endpoint, obtaining non-statistically significant results (*p* = 0.957). Table 4 summarizes the therapeutic responses of tinnitus to tDCS, based on the *p*-value of each study.

**Table 4:** Therapeutic response of tinnitus to tDCS, ordered by year of publication.

References	Scale used (primary endpoint)	Therapeutic response based on *p* value (primary endpoint)
Vanneste et al.^6^	VAS (intensity and stress)	*p* < 0.001* (AR/CL)
		*p* > 0.05 (AL/CR)
Frank et al.^11^	THI	*p* = 0.957
Garin et al.^9^	VAS (intensity and discomfort)	*p* = 0.013* (C/I)
Faber et al.^8^	VAS (annoyance)	*p* = 0.020*(A/I)
		*p* = 0.108 (D)
		*p* < 0.05*(AL/AR)
Forogh et al.^10^	CGI	*p* = 0.807
Pal et al.^7^	THI	*p* = 0.69

AR, anode on the right; AL, anode on the left; CR, cathode on the right; CL, cathode on the left; C, cathode; A, anode; I, intensity; D, discomfort; VAS, visual analog scale; THI, tinnitus handicap inventory; CGI, clinical global impression scale.

### Comparison between therapeutic response when the LTPA and DLPFC were stimulated

Among the studies that used LTPA as a stimulus site, only one study^9^ (50%) obtained some statistically significant result. Concerning the studies with stimulus in the DLPFC, two of them^6^, ^8^ (50%) obtained statistically significant results. Table 5 describes the positioning of the electrodes in each study separately and their correspondence with the International Electroencephalogram System.

**Table 5:** Positioning of the electrode in each study separately.

References	Place of stimulation	Correspondence in the international electroencephalogram system
Vanneste et al.^6^	DLPFC	F3 and F4
Pal et al.^7^	AC/PFC	T3 and T4/F3 E F4
Faber et al.^8^	DLPFC	F3 and F4
Garin et al.^9^	LTPA	Between T4 and F8
Forogh et al.^10^	LTPA	Between C3 and T5
Frank et al.^11^	DLPFC	F3 and F4

DLPFC, dorsolateral pre-frontal cortex; LTPA, left temporoparietal area; AC, auditory cortex.

## Discussion

Based on this systematic review, a sample of 602 patients was obtained, with different stimulation sites. From the evaluation criteria used by each study, there was a positive response to tDCS in 14.86% of the participants. Such positive response was obtained from two of the six participant studies (33.3%).

Compared to the only review study ever published in the literature on the subject, there was an important difference, as it shows a response to tinnitus of 39.5%.^4^ Such discrepancy may be related to the emergence of new studies already added to the present systematic review which, in turn, did not show positive responses of tDCS to tinnitus.^7^, ^10^ Additionally, the use of several methods and scales to quantify tinnitus impair result homogenization.^12^ Another impasse also related to the divergence of the results in such studies may be associated with the differences related to the definitions of tinnitus itself, as mentioned by Duijvestijing et al.^13^

Among the evaluation criteria used to measure the therapeutic response of tDCS to tinnitus, those that were successful, that is, a positive answer proved by the selected studies, were those related to the intensity and stress caused by tinnitus. The chosen scale was the visual analogue scale (VAS), which consists of asking the participant to quantify the annoyance or stress caused by the tinnitus on a score of 0–10, using an appropriate and standard ruler. However, this evaluation has often been considered superficial, since it relies on the patient's psychological and intellectual characteristics.^14^, ^15^

Thus, Figueiredo et al.,^14^ developed a comparative cross-sectional study between the VAS scale and the tinnitus handicap inventory (THI) scale, considered by many researchers to be more complex and, therefore, more effective. This study was able to identify a positive correlation between VAS and THI, that is: the higher the VAS score, the higher the THI score. Such a conclusion suggests a greater reliability of studies that apply the VAS scale in their evaluations, even considering that the THI is even more complete.

Among the stimulated areas, Vanneste et al.^6^ stimulated the bilateral dorsolateral prefrontal cortex, while the study by Garin et al.^9^ stimulated the left temporoparietal region. Other studies, such as the one by Fregni et al.^16^ had already demonstrated a positive response to stimulation on the left temporal region. Such an assertion may corroborate the differences between the therapeutic responses of the selected studies, even when the same regions were used for the stimulus.

When comparing the mean age of patients in this systematic review with reviews related to tinnitus prevalence, a similar age group, ranging from 20 to 80 years, with a mean of 55.6 years, was observed. As the age range is very large, it is possible that the causes of tinnitus in younger and older individuals may be different, which may influence the therapeutic response of tDCS. Moreover, as in this study, the larger portion of the sample comes from developed countries, and no data were available on tinnitus in patients from developing countries.^17^, ^18^

A large cross-sectional study developed in the United States by Bhatt et al.^19^ analyzed the prevalence of tinnitus using a questionnaire, as well as the intensity and annoyance caused the disease, which was answered by 75,764 inhabitants. Such a study further corroborated the prevalence of tinnitus in the population, since 9.6% of the respondents mentioned having experienced tinnitus and, of these, 56.1% of them for more than 5 years. The mean age was also similar, with this study showing a mean age of 53.1 years.

The main limitations of the present review are related to the heterogeneity of the selected studies, regarding the definition of tinnitus and also the applied scales, which results in many divergences when the results are compared. Additionally, the lack of studies in Brazil makes it more difficult to extrapolate the results found in the Brazilian population, since most of the studies occur in developed countries, with a greater literary quota on the prevalence and incidence of the disease.

Due to the heterogeneity of the studies on the subject, there is still no therapeutic response of tinnitus to tDCS. Because of the small number of studies using similar methodologies, it was also not possible to perform a meta-analysis, thus culminating in a qualitative analysis. Hence, the results found reinforce the need for more clinical trials, mainly in Brazil, to verify the efficacy of tDCS in the treatment of these patients.

However, in this systematic review, articles that demonstrated a positive response with the use of stimulation more often indicated a reduction in tinnitus intensity.

## Conclusion

Based on the present review, there is still no firm evidence of a positive therapeutic response of tinnitus to tDCS.

## Conflicts of interest

The authors declare no conflicts of interest.
